# Effects of captive and primate-focused tourism on the gut microbiome of Tibetan macaques

**DOI:** 10.3389/fmicb.2022.1023898

**Published:** 2022-10-13

**Authors:** Yingna Xia, Xiaojuan Xu, Huijuan Chen, Ran Yue, Dongpo Xia, Xi Wang, Jinhua Li, Binghua Sun

**Affiliations:** ^1^School of Resource and Environmental Engineering, Anhui University, Hefei, China; ^2^International Collaborative Research Center for Huangshan Biodiversity and Tibetan Macaque Behavioral Ecology, Anhui University, Hefei, China; ^3^School of Life Sciences, Hefei Normal University, Hefei, China; ^4^School of Life Sciences, Anhui University, Hefei, China

**Keywords:** gut microbiome, primate-focused tourism, captive, Tibetan macaque, wild

## Abstract

Documenting the effects of anthropogenic activities on the gut microbiome of wild animals is important to their conservation practices. Captivity and ecotourism are generally considered two common anthropogenic disturbances on the health of nonhuman primates. Here, we examined the divergences of gut microbiome in different environments of Tibetan macaques. Our results showed that there were no significant differences in the alpha diversity, predominant families and genera of gut microbiomes between wild and tourist groups. However, these indexes decreased significantly in the captive individuals. In addition, the significant differences of beta diversity and community compositions between wild and tourism groups also were detected. In particular, higher potential pathogenic and predicted KEGG pathway of drug resistance (antimicrobial) were detected in the gut microbiome of individuals in captive environment. Our results indicated that living in the wild are beneficial to maintaining gut microbial diversity of Tibetan macaques, while captivity environment is harmful to the health of this macaque. Exploring ways to restore the native gut microbiome and its diversity of captive individual should pay more attention to in the future studies.

## Introduction

Nonhuman primates (NHPs) are our closest living biological relatives, which can offer critical insights into the human evolution, behavior and biology, as well as the forest ecosystem health. Current information shows that the existing primates consist of 506 species in 79 genera. Unfortunately, above 60% of primate species are now threatened with extinction and above 75% have declining populations (Estrada et al., 2017). Habitat disturbance, caused by human activities, is considered the most important factor contributing to the decline in the wild primate populations (de la Torre et al., 2000). Assessing the impacts of anthropogenic disturbance on the health of wild NHPs has become one of the important goals of wild living primate conservation (Junge et al., 2011; Luo et al., 2015; Stumpf et al., 2016; Cavada et al., 2019).

Recent studies highlight that habitat disturbance can result in the loss of gut microbial diversity in the wild NHPs. For example, research on wild black howler monkeys (*Alouatta pigra*) indicates that populations of degraded habitats risk ‘double jeopardy’ from both reduced resource availability and the diversity of gut microbiome (Amato et al., 2013). Similarly, wild populations living in fragmented habitats of Udzungwa red colobus (*Procolobus gordonorum*) have a lower gut microbial diversity compared to intact habitats, which is potentially linked to a decreased ability to digest toxic plant compounds (Barelli et al., 2015). Meanwhile, populations living in the disturbed habitat, both black howler monkeys and ring-tailed lemurs (*Lemur catta*), were more enriched by potentially pathogenic microorganism (Amato et al., 2016). The gut microbiome is known to play a crucial role in host nutrition, metabolic activity, immune homeostasis and behavioral patterns (Hooper and Gordon, 2001; Archie and Theis, 2011; Nicholson et al., 2012; Wheeler et al., 2016). Understanding how the gut microbiome of wild primates is influenced by habitat disturbance presents a new study area for conservation biologists (Stumpf et al., 2016; Clayton et al., 2018; Trevelline et al., 2019).

Captivity and ecotourism are generally considered two common anthropogenic disturbances on the health of nonhuman primates. For the primates that living in captive environments, individuals usually face changes or restrictions in diet, treatments with antibiotics, increased social pressure, limited in exposure to environmental microbes, as well as exposure to human-associated microbes (McKenzie et al., 2017). Many studies have shown that these changes or restrictions are associated with the dysbiosis of the animals’ gut microecosystem, including the reduction of native gut microbes and the loss of microbial diversity (Amato et al., 2013; Clayton et al., 2016; McKenzie et al., 2017; Frankel et al., 2019), as well as increased in antibiotic resistance genes of gut microbiota (Tsukayama et al., 2018). Therefore, studies on the gut microbiome of captive individuals can inform captive management and conservation strategies for the protected animals (Stumpf et al., 2016; Trevelline et al., 2019; West et al., 2019).

Primate-focused tourism is considered as one effective strategy to achieve species conservation, financial and educational benefits for local communities (Johns, 1996; Berman and Li, 2002). As part of efforts to protect primates, invasive management methods such as translocation, provision and range restriction are often used to increase tourists’ opportunities to encounter and/or see wild primates in many primate habitat countries (Struhsaker and Siex, 1998; Berman et al., 2007). Nevertheless, a number of previous studies have shown that tourism had many detrimental effects on the health, behavior and biology of wild NHPs, including changes of activity budgets and foraging patterns (Griffiths and Schaik, 1993; Hill, 1999; de la Torre et al., 2000), increased in individual stress and intra-group competition (Maréchal et al., 2016), as well as the potential for disease transmission (Woodford et al., 2002; Devaux et al., 2019). Given host diet and stress is closely related to the composition and metabolic functions of gut microbiome (Muegge et al., 2011; Foster and McVey Neufeld, 2013; Xu et al., 2020), the gut microbiome may offer valuable insight into the effects of the tourism on primates health, nutrition, disease, as well as the conservation decisions of wild primates (Stumpf et al., 2016). To date, little is known concerning the effects of primate-focused tourism on the gut microbiomes of wild NHPs.

As a species of genus *Macaca*, the Tibetan macaque (*Macaca thibetana*) is a Near Threatened primate species endemic to east central China, which habitat in subtropical, deciduous and evergreen broad-leaved forest (Sun et al., 2010). The free-ranging with semi-provisioned group of Tibetan macaques, habitat in Mt. Huangshan, Anhui province, presents a good opportunity to assess the effects of primate-focused tourism on the gut microbiomes of wild NHPs. In 1992, local government decided to drive the group named Yulinkeng A1 (YA1), ~1 km from their natural range, to an unoccupied area. Since then, to facilitate tourists’ viewing opportunities, park staff has provided the group of ~6 kg of whole corn per day. In the present study, we compared the gut microbiomes of three groups living in different environmental settings. YA1 is a free-ranging with semi-provisioned group (Mt. Huangshan), which has long been used for primate-focused tourism. The study subjects also included a group lived in the captivity (Tong Ling City Zoo), and a wild group located some 10 km from Mt. Huangshan. We focus on the following three main questions. First, if the anthropogenic disturbance including primate-focused tourism and captivity can result in the loss of gut microbial diversity in Tibetan macaques? Second, are there significant differences in the gut microbial composition among primate-focused tourism, wild and captivity groups of Tibetan macaques? Third, what are the potential impacts of primate-focused tourism on the Tibetan macaque’s health based on the current gut microbial data? The results of this study will improve our understanding of the potential effects of anthropogenic disturbance on the primate gut microbiome.

## Materials and methods

### Study objects and samples collection

This study was conducted at three sites in southern Anhui Province, China, including Mt. Tianhu (Wild group), Mt. Huangshan (Tourism group), and the Tong Ling City Zoo (Captive group). Individuals of the tourism group were supplied 3 times a day with a total of 6–8 kgs of corn. The amount of feeding was about one third of the group’s daily food intake. Mt. Tianhu located 10 kilometers away from Mt. Huangshan. Individuals of this group get all their food from the wild. The habitats of Tibetan macaques at both Mt. Tianhu and Mt. Huangshan are evergreen broad-leaved forest and deciduous broad-leaved forest respectively, with similar flora and fauna. Individuals of the captive population in Tong Ling City Zoo were migrated from Mt. Huangshan for about 1 year during the sampling period. This group’s main diet was corn and sweet potatoes. All samples were collected from August, 2019, during a 2-week period. In total, 70 fresh fecal samples of macaques were sampled, including 26, 18 and 26 samples from the tourism, wild and captive group, respectively.

All fecal samples were stored in a sterilized tube with RNAlater (QIA-GEN, Valencia, CA, United States). Samples were transported to the laboratory of Anhui University in ice packs and stored at −80°C before DNA extraction. This research was approved by the Institutional Animal Care and Use Committee of the Anhui Zoological Society (permit number AHZS201711008). We performed all experiments in accordance with their approved guidelines and regulations, and complied with all principles of the China Animal Ethics Committee.

### DNA extraction and sequencing

To avoid contamination, we extracted DNA from the inside of each fecal sample using a QIAamp^®^ Fast DNA Stool Mini kit (Qiagen). The total DNA extracted from the fecal samples were sent to the Majorbio Bio-pharm Technology Co., Ltd. (Shanghai, China) for sequencing. The V3-V4 region of the 16S rRNA gene was amplified using primers 338F (5’-ACTCCTACGGGAGGCAGCAG-3′) and 806R (5’-GGACTACHVGGGTWTCTAAT-3′) as previously described (Mori et al., 2014). PCR reaction mixtures contained 5–100 ng of DNA template, 1 × GoTaq Green master mix, 1 M MgCl_2_, and 5 pmol of each primer. Reaction conditions include an initial 95°C for 2 min, followed by 35 cycles of 95°C for 30s, 55°C for 30s, and 72°C for 60s, and a final extension of 72°C for 5 min. After quantification step, amplicons were pooled in equal amounts, and pair-end 2 × 300 bp sequence was performed using the Illlumina Miseq platform (San Diego, CA, United States).

### Bioinformatics and statistical analysis

We trimmed raw FASTQ sequencing data for the adaptor sequence and for quality control using the sliding window approach implemented in fastp v0.19.6 (Chen et al., 2018). Sequences containing N bases were removed. FLASH v1.2.7 was used to merge overlapping paired-end reads (Magoč and Salzberg, 2011). DADA2 within Qiime 2 was used to truncate forward and reverse reads, to denoise the data, and to detect and remove chimeras (Bolyen et al., 2019). Taxonomy was assigned to amplicon sequence variants (ASV) using classify-sklearn (Naive Bayes) with the database (v.132).^1^ Qiime 2 was used to calculate Shannon diversity index, ASV richness, and unweighted and weighted UniFrac distance matrix. The sequence data has been stored in NCBI (project number is PRJNA871105).

Kolmogorov–Smirnov normality tests were used to evaluate the normal distribution of alpha diversity index and relative abundance of dominant phyla. Principal coordinates analysis (PCoA) was performed with the R packages Made4 and Vegan. Permutational multivariate ANOVA (PERMANOVA) was used to test variations in beta diversity (unweighted and weighted UniFrac distance) across the three different macaque groups using the Adonis functions in the vegan R package (Chen et al., 2012). We used Kruskal-Wallis ANOVA with Tukey’s *post-hoc* tests to test the variation in different study groups. Values of *p* were adjusted using a false discovery rate (FDR) correction. Linear discriminant analysis effect size and default options were used to determine the phylum, class, order, family and genera enriched in each study group (Segata et al., 2011). BugBase tool was used to evaluate wide-scale phenotypic properties of the gut microbiome (Ward et al., 2017). In addition, to explore the functional profiles of our data set, KEGG pathways were predicted using Phylogenetic Investigation of Communities by Reconstruction of Unobserved States (version 2; PICRUSt 2).

## Results

### General characteristics of gut microbiome profile

**Figure 1:**
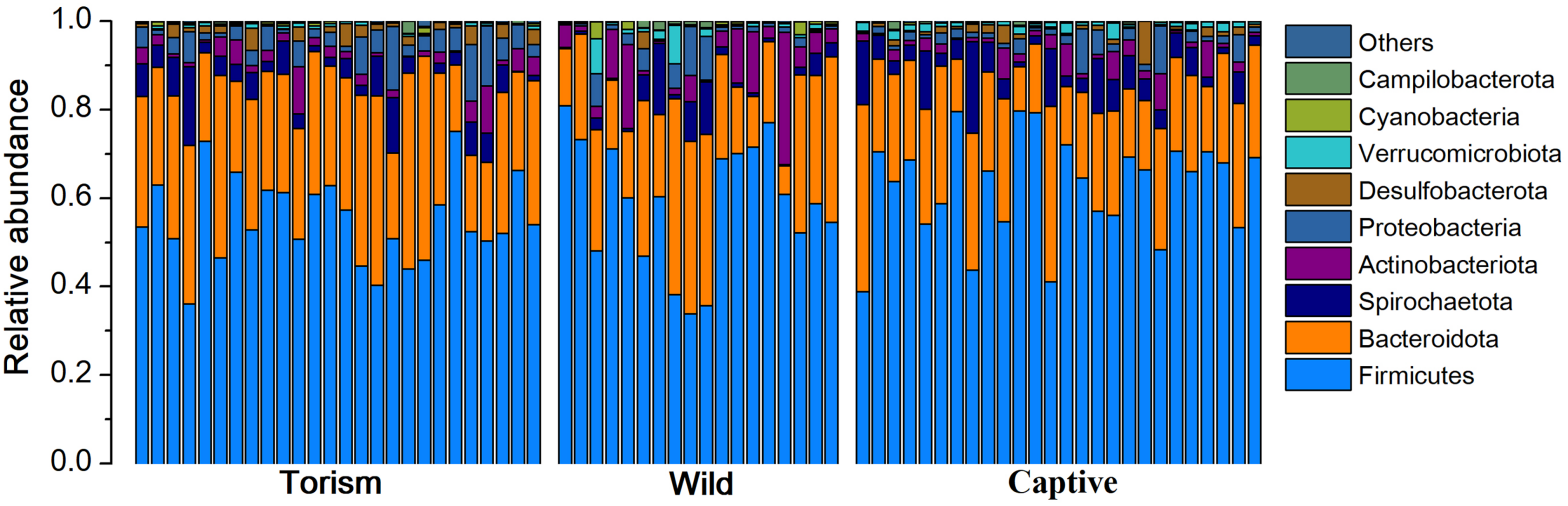
Relative abundance of fecal bacterial taxa at the phylum level. Stacked bar graphs illustrate the abundances of phyla and the x-axis represents the sample groups.

We acquired 2,063,887 high-qualities reads with 29,484 (ranging from 19,495 to 38,608 across all 70 samples) sequences per sample. Taxonomic assignment revealed 22 known bacterial phyla at 97% sequence identity. The dominant phyla were Firmicutes (x = mean ± SD, x = 58.91 ± 11.89%), Bacteroidetes (x = 25.79 ± 9.39%; Figure 1). The predominant families were Prevotellaceae (x = 17.13 ± 9.18%), Lachnospiraceae (x = 12.39 ± 9.05%) and Oscillospiraceae (x = 10.80 ± 4.10%). At the genus level, the fecal samples were dominated by *Prevotella* (x = 12.90 ± 7.89%), *UCG-005* (Oscillospiraceae; x = 5.29 ± 3.23%) and *Treponema* (x = 4.13 ± 3.81%).

### Variation of gut microbial diversity and composition among different groups

**Figure 2:**
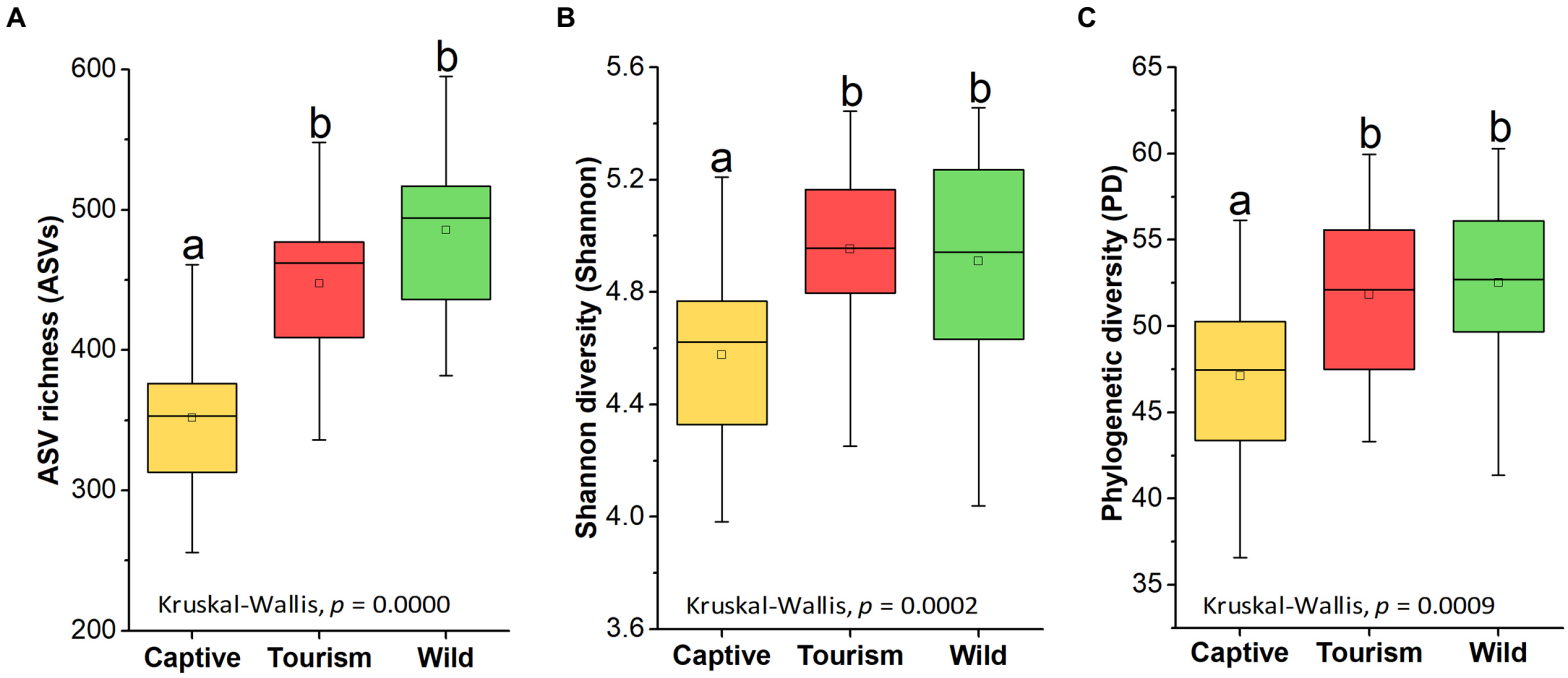
Variations on the alpha diversity in the gut microbiome among three study groups. **A**: Comparison of ASV richness. **B**: Comparison of Shannon diversity index. **C**: Comparison of PH diversity index. A Kruskal-Wallis test was used to evaluate the variation across three groups. Post-hoc tests (Tukey–Kramer test) for pairwise comparison tests (*p* values were adjusted by FDR). Letters in **(A)**, **(B)**and **(C)** represent significant differences.

Amplicon sequence variants richness (ASVs), Shannon diversity (Shannon) and Phylogenetic diversity (PD) index showed significant difference among the three study groups (Kruskal-Wallis, *F* = 34.36, df = 69, adjusted *p* < 0.001, *F* = 16.65, df = 69, adjusted *p* < 0.001 and *F* = 13.93, df = 69, adjusted *p* < 0.001). Pairwise comparison analysis showed that the ASV richness of the captive group was significantly lower which compared to the other two groups (Tukey–Kramer, Captive vs. Wild: adjusted *p* < 0.001; Captive vs. Tourism: adjusted *p* < 0.001). The same results of the other two indices also were detected, including Shannon diversity (Tukey–Kramer, Captive vs. Wild: adjusted *p* < 0.001; Captive vs. Tourism: adjusted *p* < 0.01) and Phylogenetic diversity (Tukey–Kramer, Captive vs. Wild: adjusted *p* < 0.01; Captive vs. Tourism: adjusted *p* < 0.01; Figures 2 A**–**C). However, no significant differences were detected between individuals in the tourism and wild groups for the three indexes of alpha diversity.

**Figure 3:**
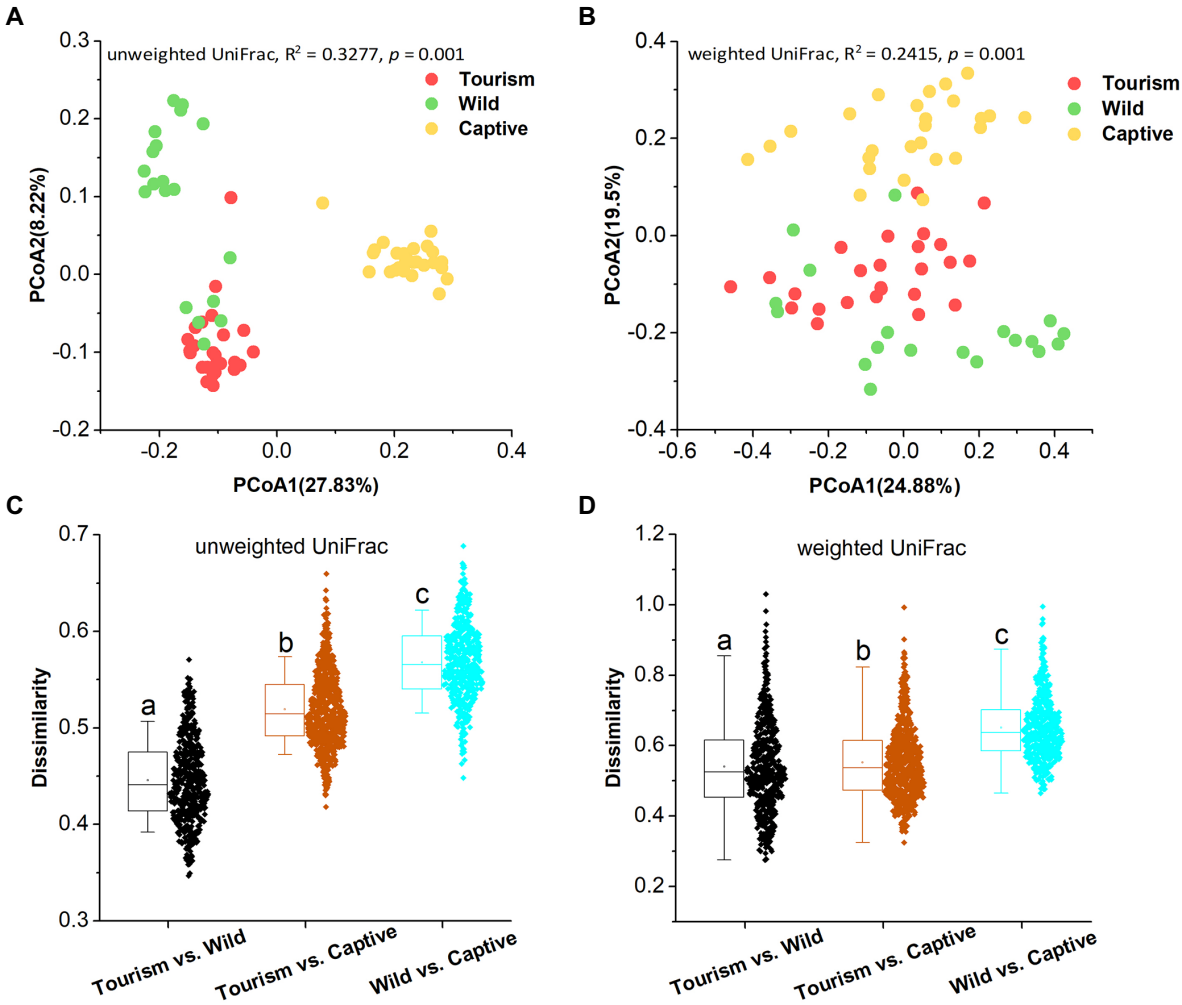
Differences in beta diversity of the gut microbiome across three study groups. **(A,B)** Differentiation of fecal microbiota structure. **A**: based on unweighted UniFrac distance, **B**: based on weighted UniFrac distance. PCoA was used to show patterns across three study groups. Adonis tests were performed on unweighted and weighted UniFrac, respectively. Significance was set at the 0.05 level. **(C,D)** Comparison of dissimilarity between Gut microbiome structures. **C**: based on unweighted UniFrac distance, **D**: based on weighted UniFrac distance. Significance was set at the 0.05 level. Letters in (C) and (D) represent significant differences.

PCoA revealed that individuals from the same group were possessed more similar microbial communities, whether based on unweighted UniFrac and weighted UniFrac dissimilarities. PERMANOVA showed the significant variation of the microbial community structures across samples from the three study groups (PERMANOVA, unweighted UniFrac, R^2^ = 0.3277, *p* = 0.001; weighted UniFrac, R^2^ = 0.2415, *p* = 0.001; Figures 3 A,B). In detail, significant differences in beta diversity between same sample types were detected based on unweighted unifrac dissimilarities (Adonis, unweighted unifrac, Captive vs. Tourism, R^2^ = 0.292, *p* = 0.001; Captive vs. Wild: R^2^ = 0.321, *p* = 0.001; Tourism vs. Wild: R^2^ = 0.137, *p* = 0.001; weighted unifrac, Captive vs. Tourism, R^2^ = 0.206, *p* = 0.001; Captive vs. Wild: R^2^ = 0.240, *p* = 0.001; Tourism vs. Wild: R^2^ = 0.115, *p* = 0.001). We found that the dissimilarity in community structures of gut microbiomes between wild and tourism was significantly lower than that between wild and captive (Wilcoxon signed-rank test, unweighted Unifrac and weighted Unifrac, *p* < 0.001; Figures 3 C,D).

### Variation of gut microbial composition among different groups

**Figure 4:**
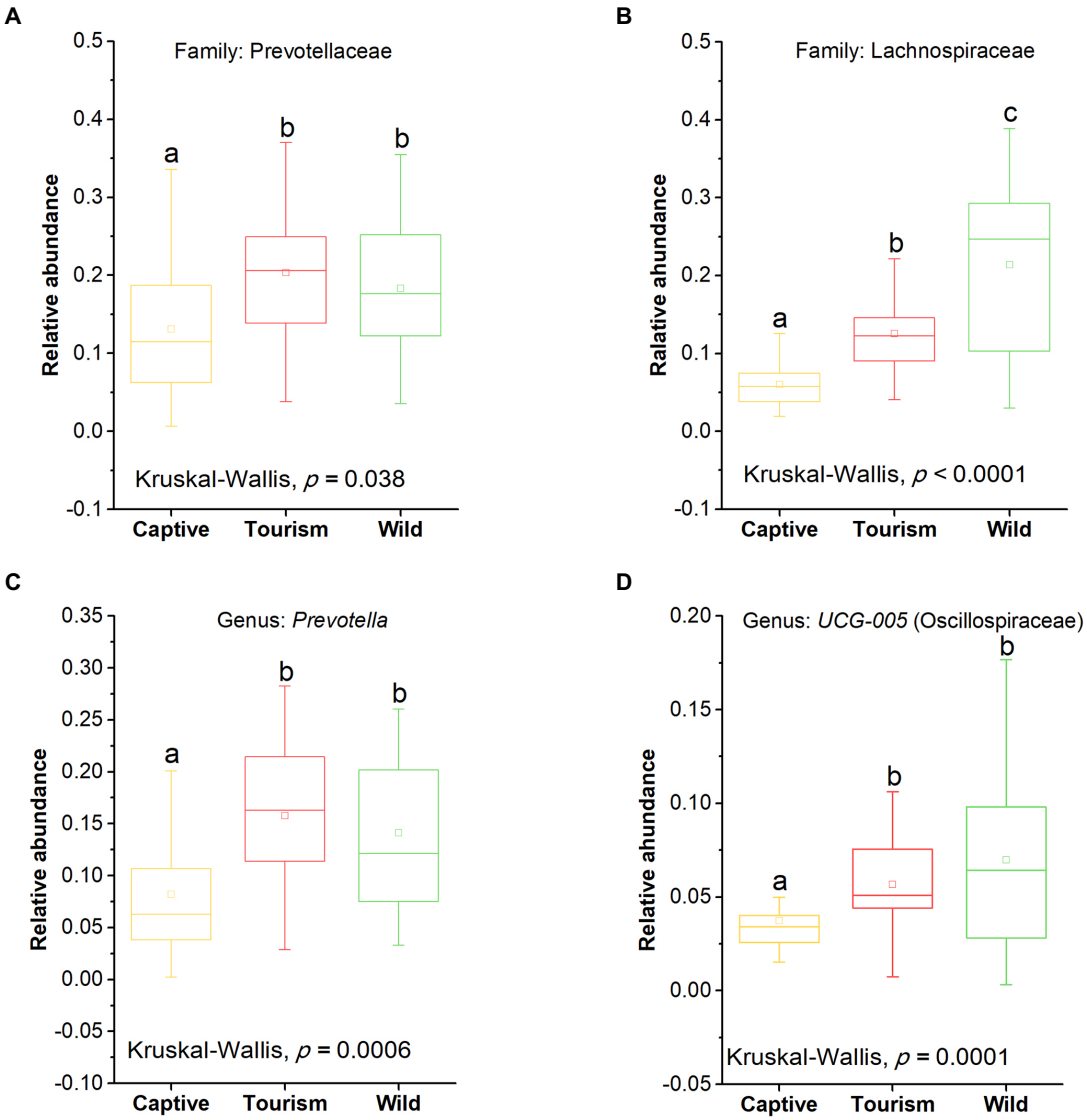
Variation in fecal bacterial taxonomy across three study groups. **(A)** and **(B)** Comparison of the predominant families. A: Prevotellaceae, B: Lachnospiraceae. **(C)** and **(D)** Comparison of the predominant genera. A: *Prevotella*, B: *UCG-005*. A Kruskal-Wallis test was used to evaluate the variation across three groups. Post-hoc tests (Tukey–Kramer test) for pairwise comparison tests (p values were adjusted by FDR). Letters in **(A)**, **(B)**, **(C)** and **(D)** represent significant differences.

The top 10 families and genera were used to evaluate the variation of gut microbial composition among different groups. We found that nine known taxa of the 10 top families showed significant variation among the three study groups (Kruskal-Wallis, *p* < 0.05; Supplementary Figure S1). In addition, eight known taxa of the 10 top genera showed significant variation among the three study groups (Kruskal-Wallis, *p* < 0.05; Supplementary Figure S2). The predominant families Prevotellaceae and Lachnospiraceae showed significant variation among the three study groups (Kruskal-Wallis, Prevotellaceae, *F* = 8.66, df = 69, adjusted *p* = 0.038; Lachnospiraceae, *F* = 30.68, df = 69, adjusted *p* < 0.001; Figures 4 A,B). *Post hoc* tests indicated that the relative abundance of Prevotellaceae in tourism group was significantly higher than for individuals in captive group (Tukey–Kramer, Captive vs. Tourism, *p* < 0.05; Captive vs. Wild, *p* > 0.05; Tourism vs. Wild, *p* > 0.05). From wild to tourism group and then to captive group, the relative abundance of Prevotellaceae was decreased significantly (Tukey–Kramer, Wild vs. Tourism, *p* < 0.01; Wild vs. Captive, *p* < 0.001; Tourism vs. Captive, *p* < 0.001). We also found that the predominant genera *Prevotella* and *UCG-005* were significant differences among the three study groups (Kruskal-Wallis, *Prevotella*, *F* = 17.24, df = 69, adjusted *p* < 0.001; *UCG-005*, *F* = 20.73, df = 69, adjusted *p* < 0.001; Figures 4 C,D). Pairwise comparison analysis showed that the *Prevotella* (Tukey–Kramer, Wild vs. Tourism, *p* > 0.05; Wild vs. Captive, *p* < 0.01; Tourism vs. Captive, *p* < 0.001) and *UCG-005* (Tukey–Kramer, Wild vs. Tourism, *p* > 0.05; Wild vs. Captive, *p* < 0.001; Tourism vs. Captive, *p* < 0.01) in the captive group were significantly lower than those of the tourism and wild groups.

**Figure 5:**
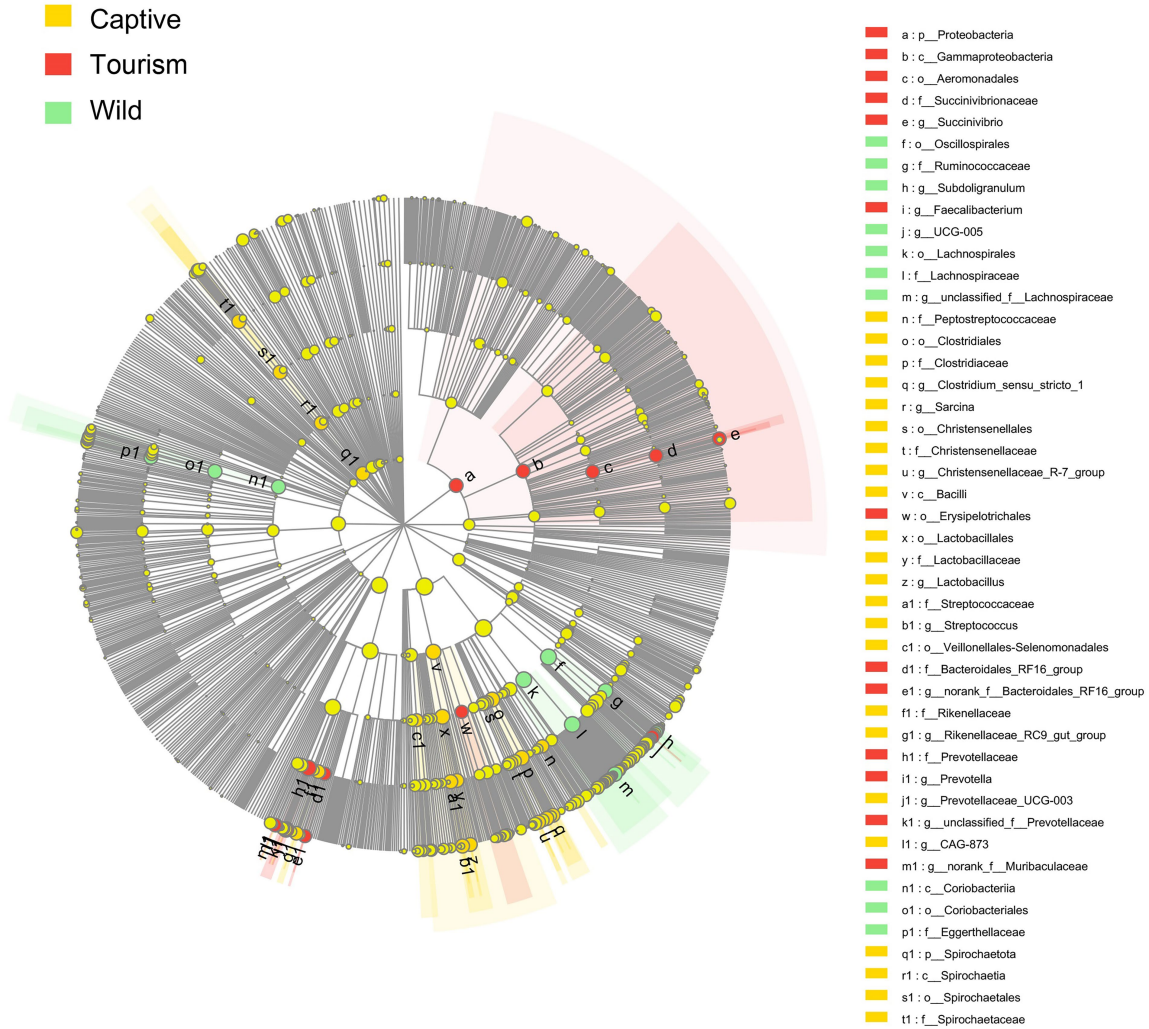
Indicators of known taxa in one of the three groups. At the genus, family, order, class, and phylum levels. Indicators identified by linear discriminant analysis effect size (LEfSe) analysis (LDA > 3, *p* < 0.05).

To explore the enriched indicators of each study group, we performed LEfSe (LDA Effect Size) analyses on the different levels of microbial taxa across samples (LDA > 4, *p* < 0.05). In total, 42 different known taxa (genus, family, order, class, and phylum levels) were significantly enriched in one of the three groups (Figure 5). Among these taxa, nine, 10, and 23 indicators were identified in wild, tourism, and captive groups, respectively. All of these known taxa were core set of the corresponding group (present in more than 90% and the average relative abundance >1% of the specific group samples). Three families (Lachnospiraceae, Ruminococcaceae and Eggerthellaceae) and two genera (*Subdoligranulum and UCG-005*) were significantly enriched in the wild group. Three families (Prevotellaceae, Succinivibrionaceae and Bacteroidales_RF16_group) and three genera (*Prevotella*, *Faecalibacterium* and *Succinivibrio*) were significantly enriched in the tourism group. Six families (Clostridiaceae, Lactobacillaceae, Christensenellaceae, Rikenellaceae, Spirochaetaceae and Peptostreptococcaceae) and eight genera (*Streptococcus*, *Sarcina*, *Clostridium_sensu_stricto_1, Christensenellaceae_R-7_group, Rikenellaceae_RC9_gut_group, Lactobacillus, CAG-873* and *Prevotellaceae_UCG-003*) were significantly enriched in the captive group.

### Variation in phenotypic properties predicted KEGG pathways of gut microbiome among different groups

**Figure 6:**
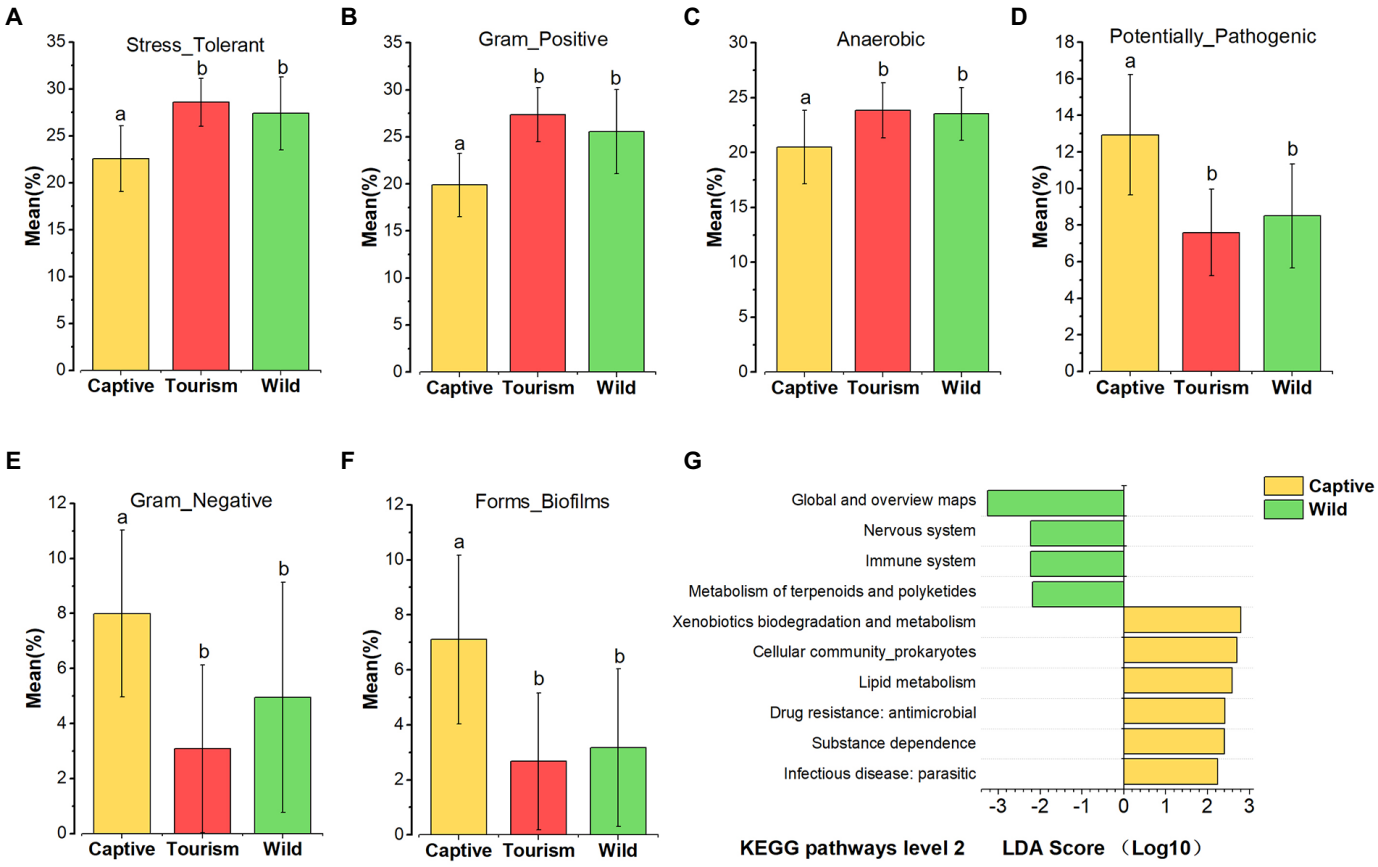
Variation in phenotypic properties and KEGG pathways of gut microbiome among different groups. **(A–F)**: Comparison of the phenotypic properties of Stress Tolerant, Gram Positive, Anaerobic, Potentially Pathogenic, Gram Negative and Forms Biofilms, respectively. A Kruskal-Wallis test was used to evaluate the variation across study groups. Post-hoc tests (Tukey–Kramer test) for pairwise comparison tests (*p* values were adjusted by Bonferroni). Significance was set at the 0.05 level. **G**: KEGG pathways at level 2. Indicators identified by linear discriminant analysis effect size (LEfSe) analysis, LDA > 2, *p* < 0.05.

Based on the BugBase tool, nine of the wide-scale phenotypic properties of the gut microbiomes were detected, including Stress Tolerant, Gram Positive, Anaerobic, Potentially Pathogenic, Contains Mobile Elements, Gram Negative, Forms Biofilms, Aerobic and Facultatively Anaerobic. Kruskal-Wallis test revealed that six phenotypic properties (Stress Tolerant, Gram Positive, Anaerobic, Potentially Pathogenic, Gram Negative and Forms Biofilms) showed significant variation on the three study groups (all the adjusted *p* < 0.001). *Post hoc* tests indicated that the proportions of predicted phenotypic including Stress Tolerant (Tukey–Kramer, Captive vs. Tourism, *p* < 0.001; Captive vs. Wild, *p* < 0.001; Tourism vs. Wild, *p* > 0.05), Gram Positive (Tukey–Kramer, Captive vs. Tourism, *p* < 0.001; Captive vs. Wild, *p* < 0.001; Tourism vs. Wild, *p* > 0.05) and Anaerobic (Tukey–Kramer, Captive vs. Tourism, *p* < 0.001; Captive vs. Wild, *p* < 0.01; Tourism vs. Wild, *p* > 0.05) were significantly lower in captive individuals than in individuals of the other two groups (Figures 6 A**–**C). In contrast, the proportions of predicted phenotypic including Potentially Pathogenic (Tukey–Kramer, Captive vs. Tourism, *p* < 0.001; Captive vs. Wild, *p* < 0.05; Tourism vs. Wild, *p* > 0.05), Gram Negative (Tukey–Kramer, Captive vs. Tourism, *p* < 0.001; Captive vs. Wild, *p* < 0.001; Tourism vs. Wild, *p* > 0.05) and Forms Biofilms (Tukey–Kramer, Captive vs. Tourism, *p* < 0.001; Captive vs. Wild, *p* < 0.001; Tourism vs. Wild, *p* > 0.05) were significantly higher in captive individuals than the tourism and wild groups (Figures 6 D**–**F).

In total, 45 KEGG pathways (level 2) were predicted by PICRUSt 2. The mean weighted nearest sequenced taxon index (NSTI) for all samples was 0.286 ± 0.056 (x = mean ± SD). We found that several predicted KEGG pathways of level 2 were enriched in one of the three study groups based on LEfSe results (LDA > 2, *p* < 0.05; Figure 6 G). Four predicted KEGG pathways were overrepresented in the wild individuals, including Metabolism of terpenoids and polyketides, Immune system, Nervous system, Global and overview maps. Additionally, six predicted KEGG pathways Infectious disease_parasitic, Substance dependence, Drug resistance (antimicrobial), Xenobiotics biodegradation and metabolism, Lipid metabolism, and Cellular community (prokaryotes) were overrepresented in captive individuals. However, we did not find any of the KEGG pathways overrepresented in the tourism group.

## Discussion

In the current study, we found that the alpha diversity of gut microbiome in the tourism group still maintain the same level as wild living individuals, which is inconsistent with previous findings that habitat disturbance can result in the loss of gut microbial diversity in the wild NHPs (Amato et al., 2013). In addition, the similarity in community structures of gut microbiomes between wild and tourism was dissimilarity lower than that between wild and captive groups. For the tourism group of Tibetan macaque used in this study, a small amount of corn provided by staff each day, but feeding from tourists were forbidden strictly. Although the group has been a subject of over 30 years for behavioral research and primate-focused tourism (Li and Kappeler, 2020), individuals of this group still maintain a direct interface with natural environment, and getting their food and water from sources which contain complexity of microbial communities (Sun et al., 2021b). Our result demonstrated that living in the wild and consuming natural diet could maintain the gut bacterial diversity of tourism group, which is consistent with our previous study on gut mycobiome in Tibetan macaques (Sun et al., 2021a).

In contrast, even though the captive group of Tibetan macaques translocated from their natural habitat only 1 year ago, the alpha diversity of this group was reduced significantly compared to the wild and tourism groups, and lower similarity in community structure of gut microbiomes between wild and captive than that between wild and tourism groups. This result is consistent with recent studies on the gut bacterial microbiome of NHPs (Clayton et al., 2016; Frankel et al., 2019; Barelli et al., 2020). Previous studies have shown that beta diversity variation was strongly influenced by host diet type and living environment (Muegge et al., 2011; Clayton et al., 2016; McKenzie et al., 2017). In addition, recent studies have reported that natural diets or releasing captive animals back into their natural habitat can help restore their native gut bacteria diversity (Schmidt et al., 2019; Liddicoat et al., 2020; Martínez-Mota et al., 2020). From wild to the captive environment, individuals of Tibetan macaques experienced a decrease in diet diversity and restricted in a direct contact with the microbes of natural environment, as well as antibiotic treatment. These changes may be responsible for the increased differentiation in community structure and decreased in diversity of captive Tibetan macaques’ gut microbiome.

We also found that the gut bacterial microbiome of Tibetan macaques in all three study groups was dominated by two phyla Firmicutes and Bacteroidetes, which also been reported in previous studies of humans and NHPs (Yatsunenko et al., 2012; Amato et al., 2013; Grieneisen et al., 2019). The two families Lachnospiraceae and Ruminococcaceae, enrichment in wild group, are highly related to the decomposition and utilization of plant diet, as well as production of the short-chain fatty acids (SCFAs; Boeckaert et al., 2008; Huws et al., 2011; Byndloss et al., 2017). In particular, we found that the Prevotellaceae and *Prevotella* were significantly enriched in the individuals of wild and tourism groups. Species of Prevotellaceae and *Prevotella* are associated with digestion of hemicellulose, pectin, starch, carbohydrate and simple sugars (Wu et al., 2011; Amato et al., 2015). These results indicated that individuals of tourism group still maintained its ability to decompose and utilize the nature plant diet. In contrast, these abilities are significantly reduced in captive Tibetan macaques. It has been shown that providing captive animals with more natural diets can help restore their native gut bacteria (Schmidt et al., 2019; Liddicoat et al., 2020; Martínez-Mota et al., 2020). Our findings, in combination with previous studies, suggest that providing more natural food may help captive Tibetan macaques recover these declining gut microbes.

Furthermore, all the wide-scale phenotypic properties of the gut microbiome, in particularly the potential pathogens, did not variation significantly between tourism and wild group. However, the two genera, including *Streptococcus* and *Sarcina*, were significantly enriched in the captive individuals of Tibetan macaque. Most members of the *Streptococcus* and *Sarcina* are potential pathogenic bacteria in humans and animals (Wyder et al., 2011; Tintara et al., 2019). The prediction results of wide-scale phenotypic properties KEGG pathways also support that the captive breeding may cause the increase of potential pathogens in gut of these macaques (Amato et al., 2016; Balasubramaniam et al., 2022). In particular, the loss of diversity in gut microbiome will increase the risk of opportunistic infection (Arrieta et al., 2014; Malard et al., 2021). In addition, KEGG pathway of drug resistance (antimicrobial), overrepresented in captive Tibetan macaques indicated that the antibiotic resistance genes (ARGs) of gut microbiomes were enriched in this group. The ARGs of a microbial community could influence the function of native bacteria and increase pathogen morbidity (Howard et al., 2003; Lin et al., 2015; Kim et al., 2020). Therefore, lower alpha diversity, enrichment in drug resistance (antimicrobial) of KEGG pathways and higher potential pathogenic bacteria among the captive Tibetan macaques have important implications the negative health consequences of captive. In the future, exploring ways to restore native gut microbiome and its diversity of captive individuals are very important for primate conservation practice.

## Conclusion

Our results provide evidence that different anthropogenic disturbances have different effects on the gut microbiome of Tibetan macaques. For the macaques used for primate ecotourism, living in the wild and consuming diverse natural diet could allow them maintain higher similarity with wild group in the alpha diversity and composition of gut microbiome including the predominant families and genera. It must be noted that the significant difference in beta diversity and community compositions between wild and tourism groups also was detected. A possible explanation is that corn provided by staff caused these changes. However, lower alpha diversity, higher KEGG pathway of drug resistance (antimicrobial) and higher potential pathogenic were detected in the gut microbiome of individuals in captive environment, which consuming a less varied diet, and limited exposure to soils and natural plants. Future studies should focus on investigating whether the changes in gut microbiome resulting from primate-focused tourism have negative effects on the health of Tibetan macaques, as well as exploring ways to restore the native gut microbiome and its diversity of captive individuals.

## Data availability statement

The datasets presented in this study can be found in online repositories. The names of the repository/repositories and accession number(s) can be found in the article/Supplementary material.

## Author contributions

BS and JL conceived and designed the experiments. BS, YX, XX, HC, RY, XW, and DX performed the experiments. BS, YX, XX, HC, and RY contributed to reagents, materials, and analysis tools. BS, YX, XX, and JL wrote the paper. All authors contributed to the article and approved the submitted version.

## Funding

This research was funded by the National Natural Science Foundation of China (grant numbers: 32171488, 31870371, and 31971404) and Scientific Research Foundation for Advanced Talents of Hefei Normal University (grant number: 2020rcjj51).

## Conflict of interest

The authors declare that the research was conducted in the absence of any commercial or financial relationships that could be construed as a potential conflict of interest.

## Publisher’s note

All claims expressed in this article are solely those of the authors and do not necessarily represent those of their affiliated organizations, or those of the publisher, the editors and the reviewers. Any product that may be evaluated in this article, or claim that may be made by its manufacturer, is not guaranteed or endorsed by the publisher.

## Supplementary material

The Supplementary material for this article can be found online at: https://www.frontiersin.org/articles/10.3389/fmicb.2022.1023898/full#supplementary-material

## Footnotes

^1^ https://www.arb-silva.de/

## References

[ref1] Amato K. R. Leigh S. R. Kent A. Mackie R. I. Yeoman C. J. Stumpf R. M.. (2015). The gut microbiota appears to compensate for seasonal diet variation in the wild black howler monkey (*Alouatta pigra*). Microb. Ecol. 69, 434–443. doi: 10.1007/s00248-014-0554-7, PMID: 25524570

[ref2] Amato K. R. Martinez-Mota R. Righini N. Raguet-Schofield M. Corcione F. P. Marini E.. (2016). Phylogenetic and ecological factors impact the gut microbiota of two Neotropical primate species. Oecologia 180, 717–733. doi: 10.1007/s00442-015-3507-z, PMID: 26597549

[ref3] Amato K. R. Yeoman C. J. Kent A. Righini N. Carbonero F. Estrada A.. (2013). Habitat degradation impacts black howler monkey (*Alouatta pigra*) gastrointestinal microbiomes. ISME J. 7, 1344–1353. doi: 10.1038/ismej.2013.16, PMID: 23486247 PMC3695285

[ref4] Archie E. A. Theis K. R. (2011). Animal behaviour meets microbial ecology. Anim. Behav. 82, 425–436. doi: 10.1016/j.anbehav.2011.05.029

[ref5] Arrieta M.-C. Stiemsma L. T. Amenyogbe N. Brown E. M. Finlay B. (2014). The intestinal microbiome in early life: health and disease. Front. Immunol. 5:427. doi: 10.3389/fimmu.2014.00427, PMID: 25250028 PMC4155789

[ref6] Balasubramaniam K. N. Aiempichitkijkarn N. Kaburu S. S. K. Marty P. R. Beisner B. A. Bliss-Moreau E.. (2022). Impact of joint interactions with humans and social interactions with conspecifics on the risk of zooanthroponotic outbreaks among wildlife populations. Sci. Rep. 12:11600. doi: 10.1038/s41598-022-15713-6, PMID: 35804182 PMC9263808

[ref7] Barelli C. Albanese D. Donati C. Pindo M. Dallago C. Rovero F.. (2015). Habitat fragmentation is associated to gut microbiota diversity of an endangered primate: implications for conservation. Sci. Rep. 5:14862. doi: 10.1038/srep14862, PMID: 26445280 PMC4595646

[ref8] Barelli C. Albanese D. Stumpf R. M. Asangba A. Donati C. Rovero F.. (2020). The gut microbiota communities of wild arboreal and ground-feeding tropical primates are affected differently by habitat disturbance. mSystems 5, e00061–e00020. doi: 10.1128/mSystems.00061-20, PMID: 32457237 PMC7253362

[ref9] Berman C. M. Li J. H. (2002). Impact of translocation, provisioning and range restriction on a Group of *Macaca thibetana*. Int. J. Primatol. 23, 383–397. doi: 10.1023/a:1013891730061

[ref10] Berman C. M. Li J. Ogawa H. Ionica C. Yin H. (2007). Primate tourism, range restriction, and infant risk among *Macaca thibetana* at Mt. Huangshan, China. Int. J. Primatol. 28, 1123–1141. doi: 10.1007/s10764-007-9199-4

[ref11] Boeckaert C. Vlaeminck B. Fievez V. Maignien L. Dijkstra J. Boon N. (2008). Accumulation of trans C18:1 fatty acids in the rumen after dietary algal supplementation is associated with changes in the Butyrivibrio community. Appl. Environ. Microbiol. 74, 6923–6930. doi: 10.1128/AEM.01473-08, PMID: 18820074 PMC2583482

[ref12] Bolyen E. Rideout J. R. Dillon M. R. Bokulich N. A. Abnet C. Al-Ghalith G. A.. (2019). QIIME 2: Reproducible, interactive, scalable, and extensible microbiome data science. Nat. Biotechnol. 37, 852–857. doi: 10.7287/peerj.preprints.27295v2, PMID: 31341288 PMC7015180

[ref13] Byndloss M. X. Olsan E. E. Rivera-Chávez F. Tiffany C. R. Cevallos S. A. Lokken K. L.. (2017). Microbiota-activated PPAR-γ signaling inhibits dysbiotic Enterobacteriaceae expansion. Science 357, 570–575. doi: 10.1126/science.aam9949, PMID: 28798125 PMC5642957

[ref14] Cavada N. Tenan S. Barelli C. Rovero F. (2019). Effects of anthropogenic disturbance on primate density at the landscape scale. Conserv. Biol. 33, 873–882. doi: 10.1111/cobi.13269, PMID: 30561170

[ref15] Chen J. Bittinger K. Charlson E. S. Hoffmann C. Lewis J. Wu G. D.. (2012). Associating microbiome composition with environmental covariates using generalized UniFrac distances. Bioinformatics 28, 2106–2113. doi: 10.1093/bioinformatics/bts342, PMID: 22711789 PMC3413390

[ref16] Chen S. Zhou Y. Chen Y. Gu J. (2018). Fastp: an ultra-fast all-in-one FASTQ preprocessor. Bioinformatics 34, i884–i890. doi: 10.1093/bioinformatics/bty560, PMID: 30423086 PMC6129281

[ref17] Clayton J. B. Gomez A. Amato K. Knights D. Travis D. A. Blekhman R.. (2018). The gut microbiome of nonhuman primates: lessons in ecology and evolution. Am. J. Primatol. 80:e22867. doi: 10.1002/ajp.22867, PMID: 29862519

[ref18] Clayton J. B. Vangay P. Huang H. Ward T. Hillmann B. M. Al-Ghalith G. A.. (2016). Captivity humanizes the primate microbiome. Proc. Natl. Acad. Sci. U. S. A. 113, 10376–10381. doi: 10.1073/pnas.1521835113, PMID: 27573830 PMC5027417

[ref19] de la Torre S. Snowdon C. T. Bejarano M. (2000). Effects of human activities on wild pygmy marmosets in Ecuadorian Amazonia. Biol. Conserv. 94, 153–163. doi: 10.1016/S0006-3207(99)00183-4

[ref20] Devaux C. A. Mediannikov O. Medkour H. Raoult D. (2019). Infectious disease risk across the growing human-non human primate Interface: a review of the evidence. Front. Public Health 7:305. doi: 10.3389/fpubh.2019.00305, PMID: 31828053 PMC6849485

[ref21] Estrada A. Garber P. A. Rylands A. B. Roos C. Li B. (2017). Impending extinction crisis of the world's primates: why primates matter. Sci. Adv. 3:e1600946. doi: 10.1126/sciadv.1600946, PMID: 28116351 PMC5242557

[ref22] Foster J. A. McVey Neufeld K.-A. (2013). Gut–brain axis: how the microbiome influences anxiety and depression. Trends Neurosci. 36, 305–312. doi: 10.1016/j.tins.2013.01.005 23384445

[ref23] Frankel J. S. Mallott E. K. Hopper L. M. Ross S. R. Amato K. R. (2019). The effect of captivity on the primate gut microbiome varies with host dietary niche. Am. J. Primatol. 81:e23061. doi: 10.1002/ajp.23061, PMID: 31713260

[ref24] Grieneisen L. E. Charpentier M. Alberts S. C. Ran B. Archie E. A. (2019). Genes, geology and germs: gut microbiota across a primate hybrid zone are explained by site soil properties, not host species. Proc. Royal Soc. B 286:20190431. doi: 10.1098/rspb.2019.0431, PMID: 31014219 PMC6501927

[ref25] Griffiths M. Schaik C. P. (1993). The impact of human traffic on the abundance and activity periods of Sumatran rain Forest wildlife. Conserv. Biol. 7, 623–626. doi: 10.1046/j.1523-1739.1993.07030623.x

[ref26] Hill D. A. (1999). Effects of provisioning on the social behaviour of Japanese and rhesus macaques: implications for socioecology. Primates 40, 187–198. doi: 10.1007/BF02557710, PMID: 23179540

[ref27] Hooper L. V. Gordon J. I. (2001). Commensal host-bacterial relationships in the gut. Science 292, 1115–1118. doi: 10.1126/science.1058709, PMID: 11352068

[ref28] Howard D. H. Scott R. D. Packard R. Jones D. (2003). The global impact of drug resistance. Clin. Infect. Dis. 36, S4–S10. doi: 10.1086/344656 12516025

[ref29] Huws S. A. Kim E. J. Lee M. R. F. Scott M. B. Tweed J. K. S. Pinloche E.. (2011). As yet uncultured bacteria phylogenetically classified as Prevotella, Lachnospiraceae incertae sedis and unclassified Bacteroidales, Clostridiales and Ruminococcaceae may play a predominant role in ruminal biohydrogenation. Environ. Microb. 13, 1500–1512. doi: 10.1111/j.1462-2920.2011.02452.x, PMID: 21418494

[ref30] Johns B. G. (1996). Responses of chimpanzees to habituation and tourism in the Kibale Forest. Uganda. Biol. Conserv. 78, 257–262. doi: 10.1016/S0006-3207(96)00044-4

[ref31] Junge R. E. Barrett M. A. Yoder A. D. (2011). Effects of anthropogenic disturbance on indri (*Indri indri*) health in Madagascar. Am. J. Primatol. 73, 632–642. doi: 10.1002/ajp.20938, PMID: 21344463

[ref32] Kim Y. Leung M. H. Y. Kwok W. Fournié G. Li J. Lee P. K. H.. (2020). Antibiotic resistance gene sharing networks and the effect of dietary nutritional content on the canine and feline gut resistome. Anim. Microb. 2:4. doi: 10.1186/s42523-020-0022-2, PMID: 33500005 PMC7807453

[ref33] Li J.-H. Kappeler P. M. (2020). “Social and life history strategies of Tibetan macaques at Mt. Huangshan” in The behavioral ecology of the Tibetan macaque (Berlin: Springer), 17–46.

[ref34] Liddicoat C. Sydnor H. Cando-Dumancela C. Dresken R. Liu J. Gellie N. J. C.. (2020). Naturally-diverse airborne environmental microbial exposures modulate the gut microbiome and may provide anxiolytic benefits in mice. Sci. Total Environ. 701:134684. doi: 10.1016/j.scitotenv.2019.134684, PMID: 31704402

[ref35] Lin J. Nishino K. Roberts M. C. Tolmasky M. Aminov R. I. Zhang L. (2015). Mechanisms of antibiotic resistance. Front. Microbiol. 6:34. doi: 10.3389/fmicb.2015.00034, PMID: 25699027 PMC4318422

[ref36] Luo J. Siemers B. M. Koselj K. (2015). How anthropogenic noise affects foraging. Glob. Chang. Biol. 21, 3278–3289. doi: 10.1111/gcb.12997, PMID: 26046451

[ref37] Magoč T. Salzberg S. L. (2011). FLASH: fast length adjustment of short reads to improve genome assemblies. Bioinformatics 27, 2957–2963. doi: 10.1093/bioinformatics/btr507, PMID: 21903629 PMC3198573

[ref38] Malard F. Vekhoff A. Lapusan S. Isnard F. D’incan-Corda E. Rey J.. (2021). Gut microbiota diversity after autologous fecal microbiota transfer in acute myeloid leukemia patients. Nat. Commun. 12:3084. doi: 10.1038/s41467-021-23376-6, PMID: 34035290 PMC8149453

[ref39] Maréchal L. MacLarnon A. Majolo B. Semple S. (2016). Primates’ behavioural responses to tourists: evidence for a trade-off between potential risks and benefits. Sci. Rep. 6:32465. doi: 10.1038/srep32465, PMID: 27628213 PMC5032226

[ref40] Martínez-Mota R. Kohl K. D. Orr T. J. Denise Dearing M. (2020). Natural diets promote retention of the native gut microbiota in captive rodents. ISME J. 14, 67–78. doi: 10.1038/s41396-019-0497-6, PMID: 31495829 PMC6908644

[ref41] McKenzie V. J. Song S. J. Delsuc F. Prest T. L. Oliverio A. M. Korpita T. M.. (2017). The effects of captivity on the mammalian gut microbiome. Integr. Comp. Biol. 57, 690–704. doi: 10.1093/icb/icx090, PMID: 28985326 PMC5978021

[ref42] Mori H. Maruyama F. Kato H. Toyoda A. Dozono A. Ohtsubo Y.. (2014). Design and experimental application of a novel non-degenerate universal primer set that amplifies prokaryotic 16S rRNA genes with a low possibility to amplify eukaryotic rRNA genes. DNA Res. 21, 217–227. doi: 10.1093/dnares/dst052, PMID: 24277737 PMC3989492

[ref43] Muegge B. D. Kuczynski J. Knights D. Clemente J. C. González A. Fontana L.. (2011). Diet drives convergence in gut microbiome functions across mammalian phylogeny and within humans. Science 332, 970–974. doi: 10.1126/science.1198719, PMID: 21596990 PMC3303602

[ref44] Nicholson J. K. Holmes E. Kinross J. Burcelin R. Gibson G. Jia W.. (2012). Host-gut microbiota metabolic interactions. Science 336, 1262–1267. doi: 10.1126/science.1223813 22674330

[ref45] Schmidt E. Mykytczuk N. Schulte-Hostedde A. I. (2019). Effects of the captive and wild environment on diversity of the gut microbiome of deer mice (Peromyscus maniculatus). ISME J. 13, 1293–1305. doi: 10.1038/s41396-019-0345-8, PMID: 30664674 PMC6474230

[ref46] Segata N. Izard J. Waldron L. Gevers D. (2011). Metagenomic biomarker discovery and explanation. Genome Biol. 12:R60. doi: 10.1186/gb-2011-12-6-r60, PMID: 21702898 PMC3218848

[ref47] Struhsaker T. T. Siex K. S. (1998). Translocation and introduction of the Zanzibar red colobus monkey: success and failure with an endangered island endemic. Oryx 32, 277–284. doi: 10.1046/j.1365-3008.1998.d01-57.x

[ref48] Stumpf R. M. Gomez A. Amato K. R. Yeoman C. J. Polk J. D. Wilson B. A.. (2016). Microbiomes, metagenomics, and primate conservation: new strategies, tools, and applications. Biol. Conserv. 199, 56–66. doi: 10.1016/J.BIOCON.2016.03.035

[ref49] Sun B. H. Li J. H. Zhu Y. Xia D. P. (2010). Mitochondrial DNA variation in Tibetan macaque (*Macaca thibetana*). Folia Zool. 59, 301–307. doi: 10.1007/s10311-015-0542-2

[ref50] Sun B. H. Xia Y. N. Davison S. Gomez A. Garber P. A. Amato K. R.. (2021b). Assessing the influence of environmental sources on the gut Mycobiome of Tibetan macaques. Front. Microb. 12:730477. doi: 10.3389/fmicb.2021.730477, PMID: 34421885 PMC8372991

[ref51] Sun B. Xia Y. Garber P. A. Amato K. R. Li J. (2021a). Captivity is associated with gut Mycobiome composition in Tibetan macaques (*Macaca thibetana*). Front. Microb. 12:665853. doi: 10.3389/fmicb.2021.665853, PMID: 33936022 PMC8085381

[ref52] Tintara S. Rice S. Patel D. (2019). Sarcina organisms: a potential cause of emphysematous gastritis in a patient with gastroparesis. Am. J. Gastroenterol. 114:859. doi: 10.14309/ajg.0000000000000124, PMID: 30848737

[ref53] Trevelline B. K. Fontaine S. S. Hartup B. K. Kohl K. D. (2019). Conservation biology needs a microbial renaissance: a call for the consideration of host-associated microbiota in wildlife management practices. Proc. Biol. Sci. 286:20182448. doi: 10.1098/rspb.2018.2448, PMID: 30963956 PMC6364583

[ref001] Tsukayama P. Boolchandani M. Patel S. Pehrsson E. C. Gibson M. K. Chiou K. L.. (2018). Characterization of wild and captive baboon gut microbiota and their antibiotic resistomes. mSystems 3:e00016-18. doi: 10.1128/mSystems.00016-18 29963641 PMC6020475

[ref54] Ward T. Larson J. Meulemans J. Hillmann B. Lynch J. Sidiropoulos D.. (2017). BugBase predicts organism-level microbiome phenotypes. bioRxiv:133462. doi: 10.1101/133462

[ref55] West A. G. Waite D. W. Deines P. Bourne D. G. Digby A. McKenzie V. J.. (2019). The microbiome in threatened species conservation. Biol. Conserv. 229, 85–98. doi: 10.1016/j.biocon.2018.11.016

[ref56] Wheeler M. L. Limon J. J. Bar A. S. Leal C. A. Gargus M. Tang J.. (2016). Immunological consequences of intestinal fungal Dysbiosis. Cell Host Microbe 19, 865–873. doi: 10.1016/j.chom.2016.05.003, PMID: 27237365 PMC4900921

[ref57] Woodford M. H. Butynski T. M. Karesh W. B. (2002). Habituating the great apes: the disease risks. Oryx 36, 153–160. doi: 10.1017/S0030605302000224

[ref58] Wu G. D. Chen J. Hoffmann C. Bittinger K. Chen Y. Y. Keilbaugh S. A.. (2011). Linking long-term dietary patterns with gut microbial enterotypes. Science 334, 105–108. doi: 10.1126/science.1208344, PMID: 21885731 PMC3368382

[ref59] Wyder A. B. Boss R. Naskova J. Kaufmann T. Steiner A. Graber H. U. (2011). *Streptococcus* spp. and related bacteria: their identification and their pathogenic potential for chronic mastitis - a molecular approach. Res. Vet. Sci. 91, 349–357. doi: 10.1016/j.rvsc.2010.09.006, PMID: 20971488

[ref60] Xu M. Wang C. Krolick K. N. Shi H. Zhu J. (2020). Difference in post-stress recovery of the gut microbiome and its altered metabolism after chronic adolescent stress in rats. Sci. Rep. 10:3950. doi: 10.1038/s41598-020-60862-1, PMID: 32127581 PMC7054252

[ref61] Yatsunenko T. Rey F. E. Manary M. J. Trehan I. Dominguez-Bello M. G. Contreras M.. (2012). Human gut microbiome viewed across age and geography. Nature 486, 222–227. doi: 10.1038/nature11053, PMID: 22699611 PMC3376388

