# Supplementary material for: Effects of captive and primate-focused tourism on the gut microbiome of Tibetan macaques
Source: Front Microbiol. 2022 Oct 13;13:1023898. doi: 10.3389/fmicb.2022.1023898 (PMC9607900; doi:10.3389/fmicb.2022.1023898)
Supplement: Supplementary file 1 [file Data_Sheet_1.docx]

Effects of captive and primate-focused tourism on the gut microbiome of Tibetan macaques - **Supplementary Material**

**Yingna Xia1,2#, Xiaojuan Xu2,3#, Huijuan Chen1,2, Ran Yue1,2, Dongpo Xia2,4, Xi Wang1,2, Jinhua Li1,2,3* and Binghua Sun1,2***


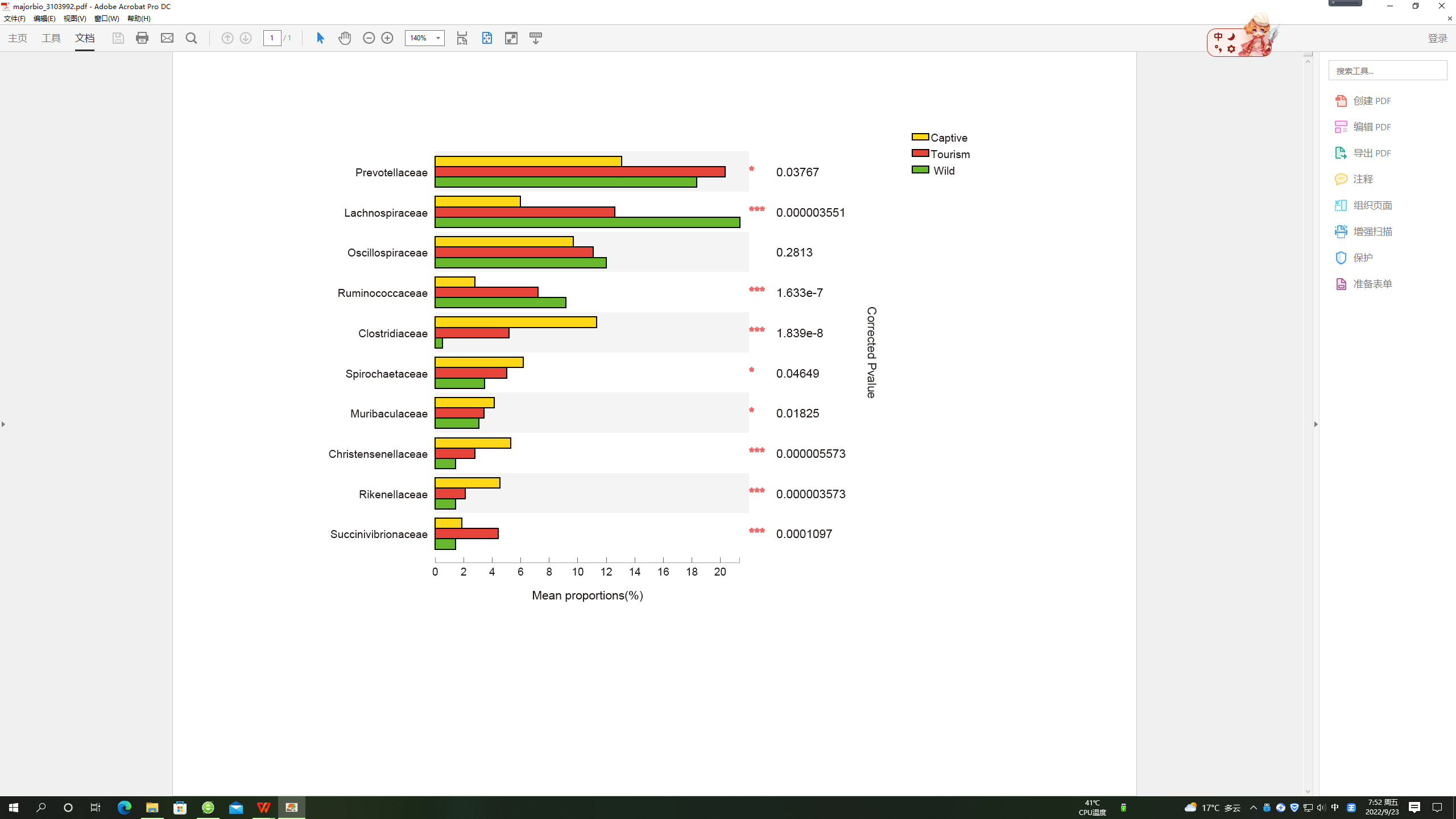


***Figure S1.* Variations of top ten families among different groups.** A Kruskal-Wallis test was used to evaluate the variation across three groups. p values were adjusted by FDR, Significance was set at the 0.05 level.


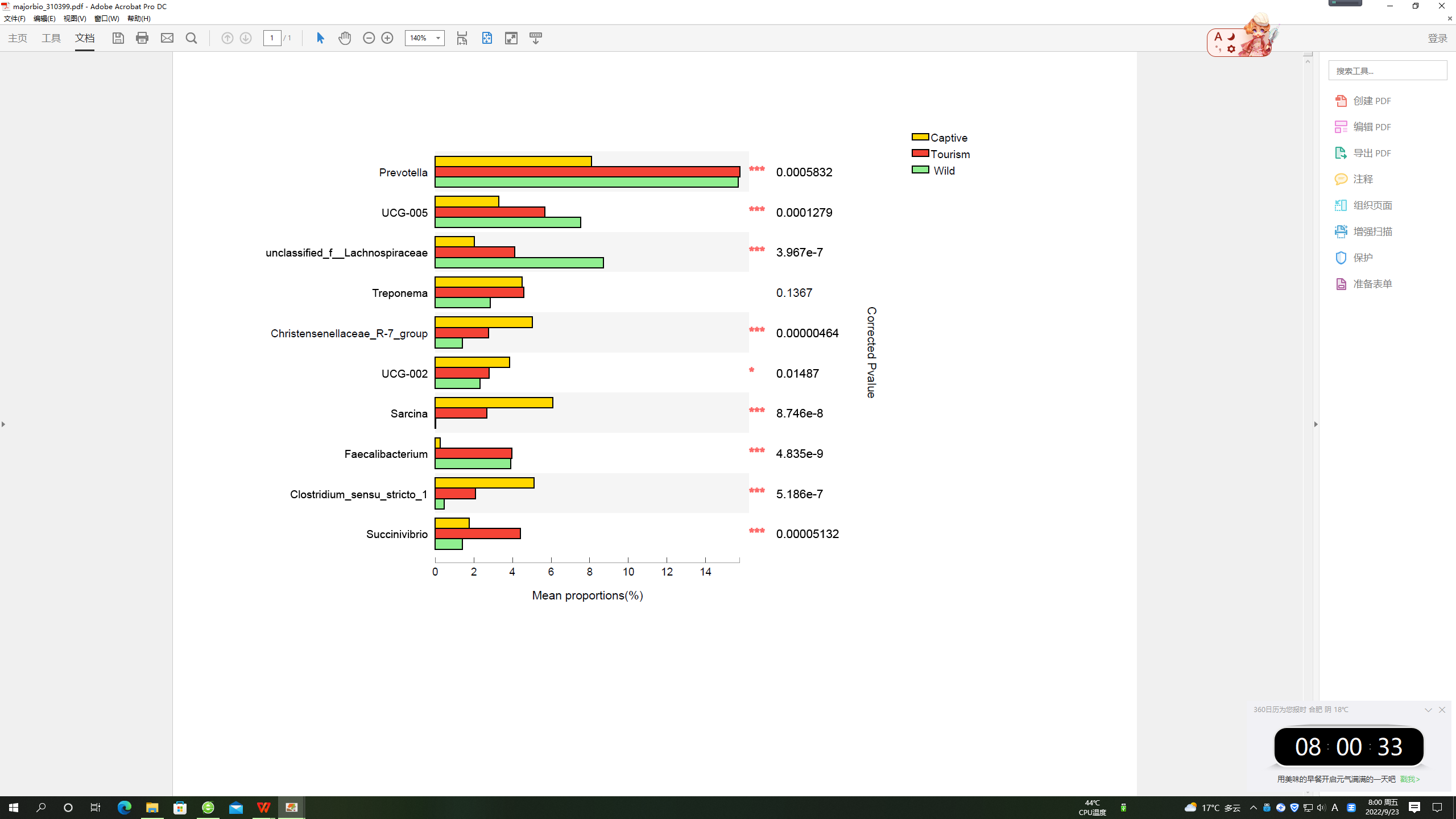


***Figure S2.* Variations of top ten genera among different groups.** A Kruskal-Wallis test was used to evaluate the variation across three groups. p values were adjusted by FDR, Significance was set at the 0.05 level.
